# Editorial: Advances in the discovery of natural molecules and their analogues against microbial infection-related biofilms

**DOI:** 10.3389/fmicb.2022.1092209

**Published:** 2022-11-25

**Authors:** Laura Quintieri, Sridhar Mani, Giovanni Lentini, Giuseppantonio Maisetta

**Affiliations:** ^1^Institute of Sciences of Food Production, National Research Council (CNR), Bari, Italy; ^2^Department of Medicine, Molecular Pharmacology and Genetics, Albert Einstein College of Medicine, The Bronx, NY, United States; ^3^Department of Pharmacy-Pharmaceutical Sciences, University of Bari Aldo Moro, Bari, Italy; ^4^Department of Translational Research and New Technologies in Medicine and Surgery, University of Pisa, Pisa, Italy

**Keywords:** biofilm, antibiotics, Quorum-Sensing, natural compounds, resistance

Bacterial biofilms are clusters of bacteria attached to a surface and embedded in a self-produced matrix. Biofilm formation can cause several infections of living tissues including wounds, lungs, and dental plaque, as well as infection of external devices such as contact lenses, prosthetic joints, and catheters (Vestby et al., 2020). The resistance of biofilm to antimicrobial agents is due to different mechanisms, such as the presence of persister cells, reduced bacterial growth rate, and limited antibiotic penetration (Ciofu et al., 2022). Despite intensive work, the therapy of biofilm-associated infections is still problematic to date. It relies on high doses of systemic antibiotic combinations to eradicate pre-formed biofilms. Nature has been always the source of inspiration for the finding of novel medicinal drugs. Natural compounds such as phytochemicals and antimicrobial peptides are promising molecules in the development of novel antibiofilm treatments (Mishra et al., 2020). Some of these are less prone to induce resistance, exhibit a wide spectrum of actions, and are suitable for chemical modification to improve pharmacological and pharmacokinetic properties. In this context, this Research Topic aimed to gather a collection of papers focused on the anti-biofilm activity of new natural molecules and synthetic analogs. A total of 13 research articles were collected disclosing new and interesting aspects of the promising approaches to prevent or eradicate microbial biofilms formed by human pathogens using plant extracts, essential oils, and nanoparticles prepared using natural compounds.

Five articles described natural compounds with antibiofilm activity against *Pseudomonas aeruginosa*, which is an opportunistic Gram-negative bacterium and a leading cause of nosocomial infections, highly contributing to morbidity and mortality of patients with cystic fibrosis (CF) or with severe burns (Reynolds and Kollef, 2021). *P. aeruginosa* readily acquires antimicrobial resistance determinants, resulting in multidrug-resistant or pan-drug-resistant strains (Reynolds and Kollef, 2021). An attempt to inhibit the biofilm formation is to quench the Quorum-Sensing (QS), which is a cell-to-cell signaling process involved in both the biofilm formation and the expression of genes associated with bacterial virulence (Rather et al., 2022; Wang et al., 2022). The QS system of *P. aeruginosa* is mainly regulated by four QS network subsystems, including lasI/lasR, rhlI/rhlR, PQS, and IQS systems (Rather et al., 2022).

In a mouse model of *P. aeruginosa*-induced acute lung infection, Tang et al. demonstrated that the phenolic compound epigallocatechin-3-gallate (EGCG), one of the richest ingredients in green tea-derived polyphenols, protected mice against *P. aeruginosa*-induced lung damage by inhibiting the virulence controlled by QS systems. In particular, EGCG decreased the expression of both QS-system genes (*las, rhl* and *pqs*) and biofilm-related genes (*pela, pila*, and *pslb*). Choi et al. observed that the Rhl QS system was selectively inhibited by curvularin, an aromatic compound isolated from the soil fungus *Phoma macrostoma*. Curvularin inhibited the production of pyocyanin and rhamnolipid and reduced the *in vivo* virulence of *P. aeruginosa* in the infection model *of Caenorhabditis elegans*.

Jia et al. observed that PQS QS system was inhibited by phthiocol (Pht), a metabolite produced by *Mycobacterium tuberculosis*. The binding of Pht and its analogs (Vitamin K1, K2 and K3) to PQS receptors of *P. aeruginosa* caused the reduction of both biofilm formation and pyocyanin production.

A standardized extract of cultured mycelium of *Lentinula edodes* (AHCC), which is available as a dietary supplement, was found by Garitaonaindia et al. to inhibit the biofilm formation of *P. aeruginosa* and to Garitaonaindia the levels of exotoxin A.

*P. aeruginosa* plays a crucial role in chronic wound infections, Di Lodovico et al. propose the *C. spinose* aqueous extract as an innovative eco-friendly strategy to prevent and control wound microbial infections. In this study authors demonstrated that such extract significantly reduced the biofilm formed by *P. aeruginosa* and *S. aureus* in the Lubbock system. This model mimics the *in vivo* microbial spatial distribution in wounds.

Although much less investigated than bacterial biofilms, also fungal biofilms play a major role in human infections (Cavalheiro and Teixeira, 2018). An increasing number of people are affected by fungal biofilm-based infections, which are resistant to the majority of currently used antifungal drugs. Only a few antifungal drugs including echinocandins and liposomal formulations of amphotericin B are available to treat biofilm-based fungal infections. In three papers on this Research Topic, the authors studied the effects of different natural compounds on fungi.

Khadke et al. demonstrated that *trans*-4-methylcinnamaldehydes which are present in the bark of trees, were able to down-regulate several genes involved in biofilm formation such as genes for hyphal development and matrix production of *Candida albicans*.

The new sodium houttuyfonate (SNH) is a compound derived from the plant *Houttuynia cordata*, an herbal drug clinically used in Asia. Zhang et al. demonstrated that SNH inhibited fungal sporulation, conidial germination, and biofilm formation. Interestingly, daily gastric gavage of SNH significantly decreased the fungal burden and local tissue damage in a mouse model of disseminated infection of *Aspergillus fumigatus*.

Wei et al. isolated from the endophytic fungus *Xylaria curta* E10 new dimeric chromanones (Paecilins) and several Paecilins showed antifungal activity against *C. albicans*.

Nanobiotechnology has gained attention in the pharmaceutical and medical fields, and the nano-sizing of antimicrobial agents seems to be a promising treatment for biofilm-related infections. In two papers on this Research Topic, antibiofilm of different chitosan formulations were studied. Chitosan (CS) is a cost-effective biopolymer suitable for preparing biocompatible, biodegradable, and non-cytotoxic nanoparticles (Kumar et al., 2022).

In the paper of Tan et al., amphotericin B (AmpB)-loaded chitosan nanoparticles (CSNP) functioned with β-1,3 glucanase (Gls), and their antibiofilm activity was evaluated against *C. albicans* biofilm *in vitro*. CSNP-AmpB-Gls inhibited biofilm formation and exhibited high efficacy in the disruption of a mature biofilm. Such nanoparticles were able to penetrate the biofilm and disassemble the biofilm matrix.

In their paper, Lin et al., combined chitosan with EDTA and the antimicrobial peptide Nile tilapia piscidin 4 (TP4). Such a formulation was tested against biofilm formed by *Gardnerella vaginalis* and *Streptococcus anginosus*, two vaginitis-associated pathogens. In addition to an antibiofilm activity *in vitro*, the TP4-chitosan formulation significantly decreased the amount of *G. vaginalis* and *S. anginosus* recovered from mice vaginal lavage, after infection with the two microorganisms. Interestingly, the TP4-chitosan formulation did not act against vaginal lactobacilli, representing an important protective component in the vaginal district.

In the paper of Lahiri et al. nanoparticles of ZnO were synthesized using the floral extract of *Clitora ternatea*, a traditionally used medicinal plant. Such nanoparticles showed stability for a long period. They were effective in the eradication of the oral biofilm formed by *Porhpyromonas gingivalis* or *Alcaligenes faecalis*, reducing the carbohydrate and protein content of the extracellular polymeric substance of biofilm.

*Melissa officinalis* is associated with phytotherapy for its sedative, antispasmodic, antimicrobial, and antioxidative activities. Yu et al. tested the essential oil of *M. officinalis* (MOEO) toward *Vibrio parahaemolyticus* that can lead to vibriosis in different species of aquatic animals, along with septicemia and gastroenteritis in humans. The Authors observed that MOEO could inhibit the biofilm formation and extracellular polysaccharide production.

Finally, Rao et al. tested SYG-180-2-2, an amide-containing compound widely present in natural products and pharmaceuticals, against methillin-resistant *Staphylococcus aureus* strains and showed that such a compound suppressed the biofilm-formation, reducing the bacterial adhesion and the production of polysaccharide intercellular adhesin.

In summary, this Research Topic provides a better understanding of the main natural and alternative components that exhibit activity in the biofilm control of pathogenic species. It is also becoming evident that the problem of biofilm-related infections can only be tackled by using interdisciplinary approaches that involve different expertise from clinicians, microbiologists, chemists, and bio-materialists. In this way, we hope that this Research Topic can generate knowledge and open ways for the construction of new strategies to combat biofilm-related infections.

## Author contributions

GM: draft preparation. LQ, SM, and GL: revision and modification. All authors contributed to the article and approved the submitted version.

## Conflict of interest

The authors declare that the research was conducted in the absence of any commercial or financial relationships that could be construed as a potential conflict of interest.

## Publisher's note

All claims expressed in this article are solely those of the authors and do not necessarily represent those of their affiliated organizations, or those of the publisher, the editors and the reviewers. Any product that may be evaluated in this article, or claim that may be made by its manufacturer, is not guaranteed or endorsed by the publisher.

## References

[B1] Cavalheiro M. Teixeira M. C. (2018). *Candida* biofilms: threats, challenges, and promising strategies. Front. Med. 5, 28. 10.3389/fmed.2018.00028 29487851 PMC5816785

[B2] Ciofu O. Moser C. Jensen P. Ø. Høiby N. (2022). Tolerance and resistance of microbial biofilms. Nat. Rev. Microbiol. 20, 621–635. 10.1038/s41579-022-00682-4 35115704

[B3] Kumar S. Dhiman R. Prudencio C. R. da Costa A. C. Vibhuti A. Leal E.. (2022). Chitosan: applications in drug delivery system. Mini Rev. Med. Chem. 10.2174/1389557522666220609102010 35692143

[B4] Mishra R. Panda A. K. De Mandal S. Shakeel M. Bisht S. S. Khan J. (2020). Natural anti-biofilm agents: strategies to control biofilm-forming pathogens. Front. Microbiol. 11:566325. 10.3389/fmicb.2020.566325 33193155 PMC7658412

[B5] Rather M. A. Saha D. Bhuyan S. Jha A. N. Mandal M. (2022). Quorum quenching: a drug discovery approach against *Pseudomonas aeruginosa*. Microbio. Res. 264, 127173. 10.1016/j.micres.2022.127173 36037563

[B6] Reynolds D. Kollef M. (2021). The epidemiology and pathogenesis and treatment of *pseudomonas aeruginosa* infections: an update. Drugs. 81, 2117–2131. 10.1007/s40265-021-01635-6 34743315 PMC8572145

[B7] Vestby L. K. Grønseth T. Simm R. Nesse L. L. (2020). Bacterial biofilm and its role in the pathogenesis of disease. Antibiotics. 9, 59. 10.3390/antibiotics9020059 32028684 PMC7167820

[B8] Wang Y. Bian Z. Wang Y. (2022). Biofilm formation and inhibition mediated by bacterial quorum sensing. Appl. Microbiol. Biotechnol. 106, 365–638. 10.1007/s00253-022-12150-3 36089638

